# Prognostic and predictive value of endothelial dysfunction biomarkers in sepsis-associated acute kidney injury: risk-stratified analysis from a prospective observational cohort of pediatric septic shock

**DOI:** 10.1186/s13054-023-04554-y

**Published:** 2023-07-03

**Authors:** Mihir R. Atreya, Natalie Z. Cvijanovich, Julie C. Fitzgerald, Scott L. Weiss, Michael T. Bigham, Parag N. Jain, Adam J. Schwarz, Riad Lutfi, Jeffrey Nowak, Geoffrey L. Allen, Neal J. Thomas, Jocelyn R. Grunwell, Torrey Baines, Michael Quasney, Bereketeab Haileselassie, Matthew N. Alder, Stuart L. Goldstein, Natalja L. Stanski

**Affiliations:** 1 Division of Critical Care Medicine, MLC2005, Cincinnati Children’s Hospital Medical Center and Cincinnati Children’s Research Foundation, 3333 Burnet Avenue, Cincinnati, OH 45229 USA; 2 Department of Pediatrics, University of Cincinnati College of Medicine, Cincinnati, OH 45267 USA; 3 UCSF Benioff Children’s Hospital Oakland, Oakland, CA 94609 USA; 4 Children’s Hospital of Philadelphia, Philadelphia, PA 19104 USA; 5 Akron Children’s Hospital, Akron, OH 44308 USA; 6 Texas Children’s Hospital, Baylor College of Medicine, Houston, TX 77030 USA; 7 Children’s Hospital of Orange County, Orange, CA 92868 USA; 8 Riley Hospital for Children, Indianapolis, IN 46202 USA; 9 Children’s Hospital and Clinics of Minnesota, Minneapolis, MN 55404 USA; 10 Children’s Mercy Hospital, Kansas City, MO 64108 USA; 11 Penn State Hershey Children’s Hospital, Hershey, PA 17033 USA; 12 Children’s Healthcare of Atlanta at Egleston, Atlanta, GA 30322 USA; 13 University of Florida Health Shands Children’s Hospital, Gainesville, FL 32610 USA; 14 CS Mott Children’s Hospital at the University of Michigan, Ann Arbor, MI 48109 USA; 15 Lucile Packard Children’s Hospital Stanford, Palo Alto, CA 94304 USA; 16 Division of Nephrology, Cincinnati Children’s Hospital Medical Center and Cincinnati Children’s Research Foundation, Cincinnati, OH 45229 USA

**Keywords:** Sepsis, Septic shock, Sepsis-associated acute kidney injury, Endothelial dysfunction, Precision medicine, Biomarkers

## Abstract

**Background:**

Sepsis-associated acute kidney injury (SA-AKI) is associated with high morbidity, with no current therapies available beyond continuous renal replacement therapy (CRRT). Systemic inflammation and endothelial dysfunction are key drivers of SA-AKI. We sought to measure differences between endothelial dysfunction markers among children with and without SA-AKI, test whether this association varied across inflammatory biomarker-based risk strata, and develop prediction models to identify those at highest risk of SA-AKI.

**Methods:**

Secondary analyses of prospective observational cohort of pediatric septic shock. Primary outcome of interest was the presence of ≥ Stage II KDIGO SA-AKI on day 3 based on serum creatinine (D3 SA-AKI SCr). Biomarkers including those prospectively validated to predict pediatric sepsis mortality (PERSEVERE-II) were measured in Day 1 (D1) serum. Multivariable regression was used to test the independent association between endothelial markers and D3 SA-AKI SCr. We conducted risk-stratified analyses and developed prediction models using Classification and Regression Tree (CART), to estimate risk of D3 SA-AKI among prespecified subgroups based on PERSEVERE-II risk.

**Results:**

A total of 414 patients were included in the derivation cohort. Patients with D3 SA-AKI SCr had worse clinical outcomes including 28-day mortality and need for CRRT. Serum soluble thrombomodulin (sTM), Angiopoietin-2 (Angpt-2), and Tie-2 were independently associated with D3 SA-AKI SCr. Further, Tie-2 and Angpt-2/Tie-2 ratios were influenced by the interaction between D3 SA-AKI SCr and risk strata. Logistic regression demonstrated models predictive of D3 SA-AKI risk performed optimally among patients with high- or intermediate-PERSEVERE-II risk strata. A 6 terminal node CART model restricted to this subgroup of patients had an area under the receiver operating characteristic curve (AUROC) 0.90 and 0.77 upon tenfold cross-validation in the derivation cohort to distinguish those with and without D3 SA-AKI SCr and high specificity. The newly derived model performed modestly in a unique set of patients (*n* = 224), 84 of whom were deemed high- or intermediate-PERSEVERE-II risk, to distinguish those patients with high versus low risk of D3 SA-AKI SCr.

**Conclusions:**

Endothelial dysfunction biomarkers are independently associated with risk of severe SA-AKI. Pending validation, incorporation of endothelial biomarkers may facilitate prognostic and predictive enrichment for selection of therapeutics in future clinical trials among critically ill children.

**Graphical abstract:**

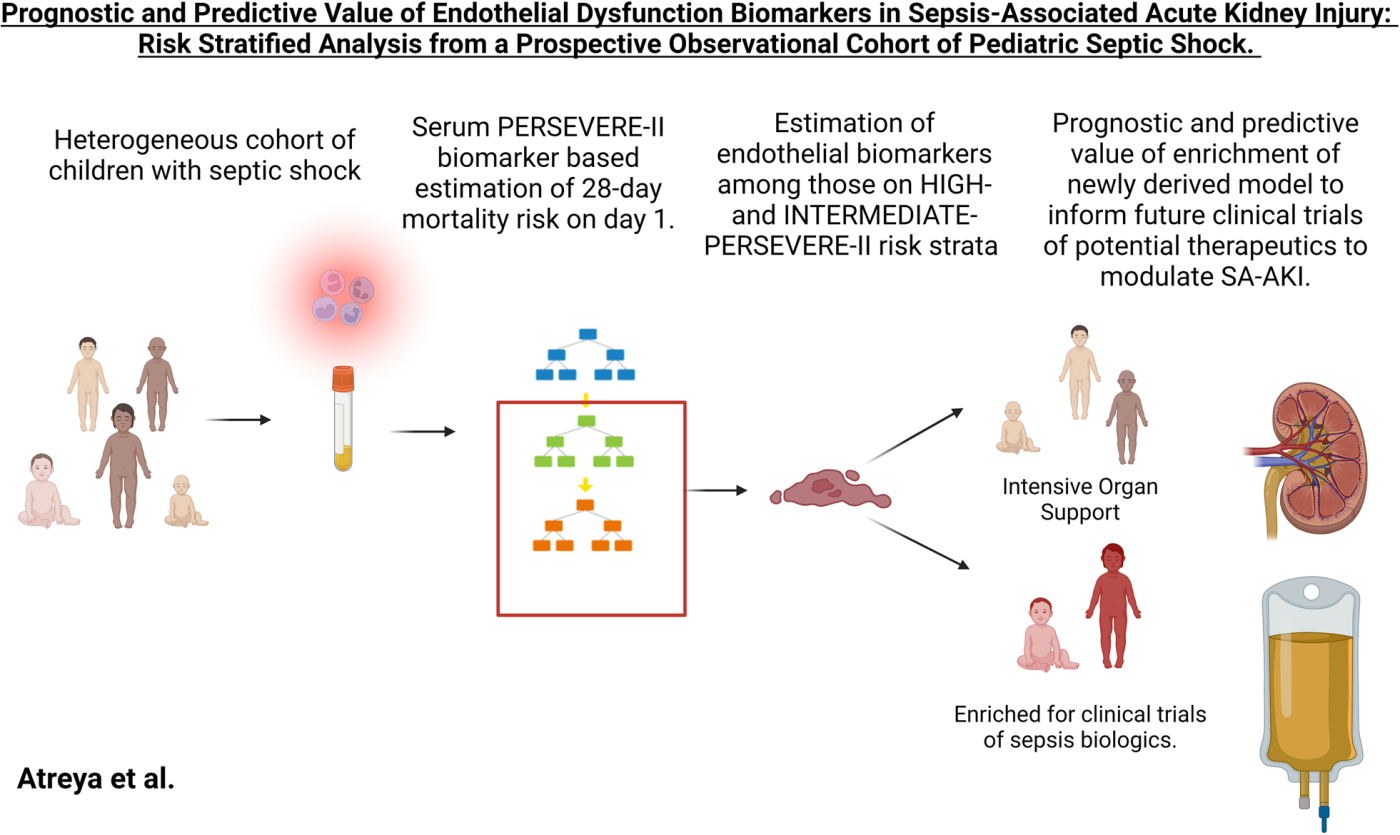

**Supplementary Information:**

The online version contains supplementary material available at 10.1186/s13054-023-04554-y.

## Introduction

Sepsis-associated acute kidney injury (SA-AKI) is common among critically ill adults and children admitted to intensive care units (ICU), affecting up to half of patients with septic shock [1–6]. Further, persistent SA-AKI is associated with high mortality rates, ranging from 30 to 70% [1–5], and new functional morbidity among affected patients [7]. Unfortunately, there are no disease modifying therapies available for SA-AKI, forcing clinicians to rely solely on supportive care measures such as continuous renal replacement therapy (CRRT) to treat affected patients. Given the substantial disease burden associated with SA-AKI, there remains a dire need to identify at-risk patients and those who may have a biological predisposition to respond to targeted therapies.

Dysregulated systemic inflammatory response to infection is undoubtedly a key contributor to the development of SA-AKI [8]. In children specifically, we have previously demonstrated that the updated Pediatric Sepsis Biomarker Risk Model (PERSEVERE-II)—a prospectively validated prognostic enrichment tool to estimate mortality risk that incorporates pediatric sepsis-specific inflammatory biomarkers and platelet count [9, 10]—is highly predictive of severe SA-AKI and its sequelae [11]. Of note, the incidence of SA-AKI and use of CRRT were higher, while renal recovery was lower, among children with high- or intermediate-PERSEVERE-II mortality strata relative to those with low- risk [11].

Microcirculatory changes including increased vascular permeability with interstitial edema and resultant tubular epithelial hypoxia are thought to be a pathognomonic feature of SA-AKI [8]. Translational studies among critically ill adults corroborate this important role of endothelial dysfunction in SA-AKI [12–15]. More recently, studies evaluating subphenotypes of SA-AKI in critically ill adults have demonstrated worse outcomes among those with both systemic inflammation and endothelial dysfunction, with subphenotype assignment carrying prognostic and potential therapeutic implications [16, 17]. However, such data are lacking among critically ill children, who may manifest age-related differences in sepsis pathobiology [18].

Accordingly, we sought to assess the association between markers of endothelial dysfunction and Day 3 (D3) SA-AKI among children with septic shock. Further, we conducted analyses to test whether the association between endothelial biomarkers and D3 SA-AKI risk differed across PERSEVERE-II mortality risk strata. Finally, we derived and validated endothelial biomarker-based models to estimate the risk of D3 SA-AKI among prespecified subgroups, based on PERSEVERE-II mortality risk.

## Methods

### Study design and patient selection

The study protocol was approved by Institutional Review Boards of participating institutions [10, 19, 20]. Briefly, patients under the age of 18 years were recruited from multiple pediatric ICUs (PICU) across the USA between 2003 and 2019. There were no study-related interventions except for blood draws. Clinical and laboratory data were available between Day 1 through 7, including platelet counts on Day 1 (D1). Baseline illness severity among patients was determined by pediatric risk of mortality (PRISM-III) score [21]. Inclusion criteria for this study were 1) patients meeting pediatric-specific consensus criteria for septic shock [22]. Exclusion criteria included (1) patients with pre-existing kidney disease (*n* = 60), (2) lack of serum creatinine (SCr) data on day 3 of septic shock (*n* = 229), and (3) those with no endothelial dysfunction marker data on D1 (*n* = 483). Severe SA-AKI was defined using serum creatinine (SCr) criteria as per Kidney Disease Improving Global Outcomes (KDIGO) stage 2 AKI or higher [23], which corresponds to a ≥ twofold increase in SCr relative to baseline. Baseline SCr values were unknown for all patients in the cohort, and thus were imputed using their calculated body surface area (m^2^) and an eGFR of 120 ml/min per 1.73 m^2^, as validated in the literature [24, 25]. Urine output data were not available for all patients in the cohort and therefore not included. All patients receiving RRT were considered to have severe AKI. The primary outcome of interest was the presence of severe SA-AKI, based on serum creatinine criteria alone, on day 3 of septic shock—a clinically relevant time point in AKI research [26]. The outcome is henceforth annotated as Day 3 SA-AKI SCr. Secondary outcomes included mortality at day 7 and day 28, complicated course (a composite of death during study period or the persistence of 2 or more organ dysfunctions on day 7 of septic shock), PICU length of stay (LOS), PICU free days—calculated by subtracting PICU LOS from a theoretical maximum of 28 days, and the use of CRRT.

### PERSEVERE-II based risk stratification

PERSEVERE-II mortality probability and risk strata were determined, according to published methods [9]. Briefly, Interleukin-8 (IL-8), Heat shock protein 70 kDA (HSP70), C-C Chemokine ligand 3 (CCL3), C-C Chemokine ligand 4 (CCL4), Granzyme B (GZMB), Interleukin-1 α (IL-1a), and Matrix metallopeptidase 8 (MMP8) were previously measured in serum collected on D1. Classification and Regression Tree (CART) analyses were used to derive a mortality probability risk score (0.000–0.999) using R software (version 4.2.2). Patients were subsequently classified as low risk (mortality probability score range ≤ 0.019), intermediate risk (mortality probability score range > 0.019 to ≤ 0.300), or high risk (mortality probability score range > 0.300).

### Serum biomarkers of endothelial dysfunction

Concentrations (in pg/mL) of soluble thrombomodulin (sTM), Angiopoietin-1 (Angpt-1), Angiopoietin-2 (Angpt-2), tyrosine kinase with immunoglobulin-like loops and epidermal growth factor homology domains-2 (Tie-2), intercellular adhesion molecule-1 (ICAM-1), Vascular Cell Adhesion Molecule-1 (VCAM-1), and Platelet Endothelial Cell Adhesion Molecule (PECAM-1) were measured in serum collected on day 1 of septic shock by Luminex assays (R&D Systems, MN), as previously published [20].

### Statistical analyses

Minitab Software (PA, USA, version 21.1.0) was used for data analyses. GraphPad Prism (CA, USA, version 9) was used to generate figures. Demographic data were summarized with percentages or median with interquartile ranges. Differences between groups were determined by *χ*^2^ test for categorical variables and by nonparametric Kruskal–Wallis H test for continuous variables. One-way analysis of variance (ANOVA) with Dunnett’s test for correction for multiple comparisons was used when comparing differences across mortality risk strata. Univariate and multivariable logistic regression with backward elimination of predictor variables (*α* < 0.05) was used to test the association between predictor variables and risk of D3 SA-AKI SCr. The latter model was adjusted for patient age, PRISM-III score, and PERSEVERE-II mortality probability score. General linear models were used to test the variation in endothelial dysfunction markers according to D3 SA-AKI SCr status, PERSEVERE-II mortality class, and an interaction term for D3 SA-AKI SCr X PERSEVERE-II mortality class. A p value of 0.05 was used to test for statistical significance.

### Risk prediction modeling

Multivariable logistic regression models incorporating biomarkers selected in the previous steps and presence of severe SA-AKI on day 1 (D1 SA-AKI SCr) were used to predict risk of D3 SA-AKI SCr across the entire cohort, and predefined subgroups including (1) a composite of PERSEVERE-II high- and intermediate-mortality risk strata and (2) PERSEVERE-II low-risk mortality risk strata alone. These subgroups were defined a priori as the number of patients deemed to be high- or intermediate-mortality risk was expected to be relatively low. Area under the receiver operating characteristic curve (AUROC) and diagnostic test characteristics are presented for training and fivefold cross-validation in the derivation cohort.

We subsequently derived a new CART model to optimize risk prediction among the subset of patients with high- or intermediate-PERSEVERE-II mortality risk using published approaches [20]. Briefly, models were weighted to match sample frequencies and within *K* = 1 standard error of minimum misclassification cost was chosen to select the optimal tree. Class probability method and tenfold cross-validation was used for CART analyses. Patients were categorized as high- versus low- D3 SA-AKI SCr risk categories based on output of the model.

### Validation in a unique set of patients

We tested the independent performance of the newly derived risk model, henceforth referred to as PERSEVERENCE SA-AKI model, in a unique set of patients with existing PERSEVERE-II biomarker data and newly measured endothelial markers. We compared the presence of D3 SA-AKI SCr among patients categorized as having high versus low risk of D3 SA-AKI SCr in the hold-out validation cohort using *χ*^2^ test. The R code for our model is provided in the supplement for the purposes of external validation.

## Results

**Fig. 1:**
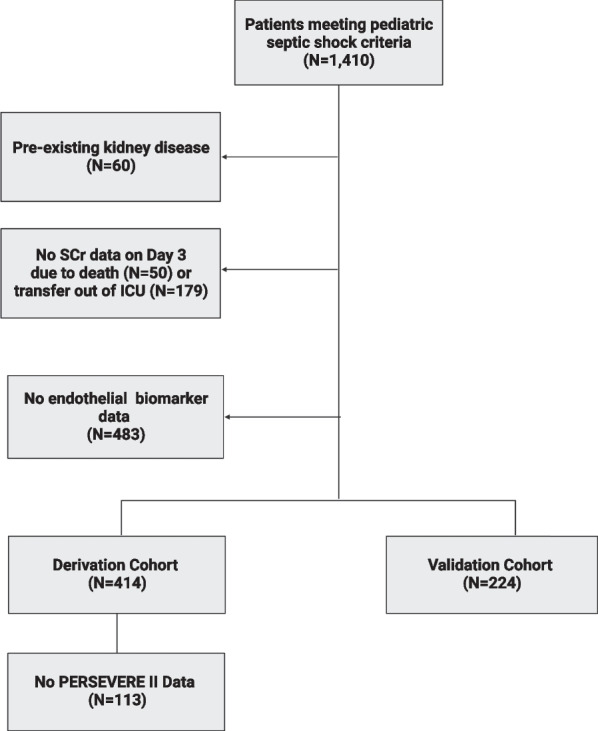
Flow diagram demonstrating inclusion and exclusion of patients in the cohort. Abbreviations: Serum Creatinine (SCr). Pediatric Sepsis Biomarker Risk Model (PERSEVERE-II)

A total of 414 patients with pediatric septic shock were included in the derivation cohort and 224 patients were included in the validation cohort, as shown in Fig. 1. One hundred and forty patients (33.8%) and 74 patients (33.0%) had D3 SA-AKI SCr in the derivation and validation cohorts, respectively. The demographic, clinical characteristics, and outcomes of patients with and without D3 SA-AKI SCr in the derivation cohort are shown in Table 1. Patients who had D3 SA-AKI SCr were younger and had higher illness severity on day 1 of septic shock. All secondary outcomes including mortality, complicated course, and need for CRRT on day 7 were higher among those with D3 SA-AKI SCr. A total of 301 patients in the derivation cohort had PERSEVERE-II biomarker data to estimate mortality probability and categorize patients according to mortality risk strata. Differences in clinical outcomes across low-, intermediate-, and high-risk PERSEVERE-II strata are presented in Additional file 1.

**Table 1:** Demographic data and clinical characteristics in the derivation cohort among patients with and without D3 SA-AKI SCr

Variable	D3 SA-AKI SCr (*n* = 140)	No D3 SA-AKI SCr (*n* = 274)	*p* value
Age (years)	2.4 (0.9, 6.7)	4.1 (1.7, 7.7)	0.003
Sex, female (%)	59 (42.2%)	131 (47.8%)	0.274
*Race (self-identified)*			
White/Caucasian	102 (72.9%)	203 (74.1%)	0.947
Black/African American	18 (12.9%)	35 (12.8%)	
Other	20 (14.2%)	36 (13.1%)	
*Ethnicity*			
Hispanic or Latino	25 (17.9%)	43 (15.7%)	0.485
PRISM-III	15 (9, 21)	11 (6, 15)	< 0.001
28-day mortality	28 (20.0%)	7 (2.6%)	< 0.001
Complicated course	94 (67.2%)	55 (20.1%)	< 0.001
PICU LOS	11 (7, 20)	7 (3, 12)	< 0.001
PICU free days	17 (8, 21)	21 (16, 25)	< 0.001
Day 7 CRRT*	29/120 (24.2%)	1/267 (0.01%)	< 0.001

*Adjusted for mortality prior to day 7

### Independent association of markers of endothelial dysfunction with risk of D3 SA-AKI

**Fig. 2:**
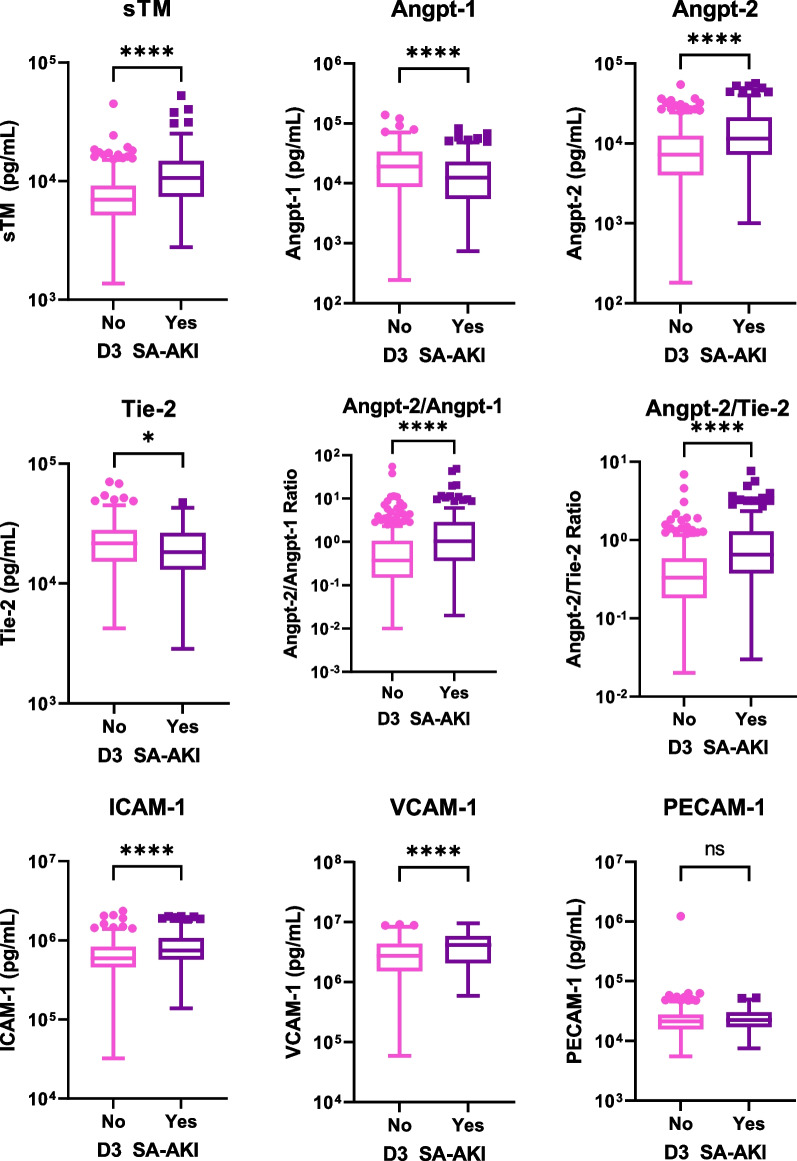
Box and whisker plots of concentrations (pg/mL) of endothelial dysfunction marker concentrations among patients with and without Day 3 sepsis-associated acute kidney injury based on serum creatinine (D3 SA-AKI SCr). *Y* axis is depicted in log scale. Asterisk * indicate a *p* value of 0.01. **** indicate a *p* value of < 0.0001

Figure 2 shows markers of endothelial dysfunction among those with and without D3 SA-AKI SCr in the derivation cohort. Concentrations of all endothelial markers tested, except PECAM-1, differed between comparison groups of interest; sTM, Angpt-2, Angpt-2/Angpt-1 ratio, Angpt-2/Tie-2 ratio, VCAM-1, and ICAM-1 were higher; Angpt-1 and Tie-2 were lower among those with D3 SA-AKI SCr relative to those without. The univariate associations between predictor variables and the risk of D3 SA-AKI are shown in Additional file 2. Results of multivariable logistic regression analyses to test the association between markers of endothelial dysfunction (log10 transformed) and risk of D3 SA-AKI SCr are presented in Table 2. Only sTM, Angpt-2, and Tie-2 were independently associated with risk of D3 SA-AKI SCr. The adjusted odds of D3 SA-AKI SCr was 18.7 (95%CI 3.2, 93.2) for each log10 fold increase in sTM, 2.81 (95%CI 1.1, 7.2) for each log10 fold increase in Angpt-2, and 0.20 (95%CI 0.05, 0.77) for each log10 fold increase in Tie-2. The association between markers of endothelial dysfunction and presence of D3 SA-AKI SCr varied across PERSEVERE-II mortality risk strata as shown in Additional file 3. Of note, Tie-2 concentrations and Angpt-2/Tie-2 ratios were influenced by the interaction between the presence of D3 SA-AKI SCr and high- and intermediate-mortality risk strata.

**Table 2:** Multivariable logistic regression analysis to test the association between D3 SA-AKI and endothelial dysfunction markers in the derivation cohort

Term	Coefficients (SE)	*p* value	Adj odds ratio	AUROC
Age (in years)	− 0.04 (0.03)	0.139	0.95 (0.89, 1.01)	0.76
PRISM-III	0.04 (0.02)	0.022	1.04 (1.01, 1.07)	
PERSEVERE-II mortality probability*	− 0.02 (0.12)	0.842	0.97 (0.77, 1.23)	
sTM (Log10)	2.93 (0.82)	< 0.001	18.7 (3.8, 93.2)	
Angpt-2 (Log10)	1.03 (0.48)	0.022	2.8 (1.1, 7.2)	
Tie-2 (Log10)	− 1.60 (0.68)	0.019	0.20 (0.05, 0.77)	

All endothelial dysfunction markers were considered in this model and backward elimination with an alpha of 0.05 was used to select variables

*AUROC*, Area under the receiver operating characteristic curve

*The raw PERSEVERE-II mortality probability was transformed by a factor of 10 for logistic regression analyses

### Endothelial dysfunction marker-based D3 SA-AKI risk prediction models perform better among patients belonging to high- or intermediate-PERSEVERE-II mortality risk strata

Performance characteristics of multivariable logistic regression models predictive of D3 SA-AKI SCr which included the presence of severe D1 SA-AKI, sTM, Angpt-2, Tie-2, and Angpt-2/Tie-2 ratio in the training and cross-validation test sets are presented in Table 3. The AUROC for this model was 0.78 in the training set and 0.77 upon fivefold cross-validation across the entire cohort. When restricted to those with high- or intermediate-PERSEVERE-II mortality risk, the model had better performance with AUROCs of 0.88 and 0.85 upon cross-validation, with sensitivity of 77.1% and 72.9% and specificity of 81.6% in both training and test sets. In comparison, the AUROCs were 0.77 and 0.73 among patients with low-mortality risk. In the low PERSEVERE-II mortality risk group, the model had high specificity but low sensitivity.

**Table 3:** Test characteristics of endothelial dysfunction marker-based multivariable logistic regression model to estimate risk of D3 SA-AKI in the entire cohort and across predefined groups based on PERSEVERE-II mortality risk

	Training set	Test set
*Entire cohort*		
AUROC	0.78	0.77
True positive, *n*	52	51
False negative,* n*	51	52
False positive, *n*	29	30
True negative, *n*	165	164
Sensitivity %	50.5%	49.5%
Specificity %	85.1%	84.5%
False positive rate (%)	14.9%	15.5%
False negative rate	49.5%	50.5%
*High and intermediate risk P-II strata*		
AUROC	0.88	0.85
True positive, *n*	37	35
False negative, *n*	11	13
False positive, *n*	9	9
True negative, *n*	40	40
Sensitivity %	77.1%	72.9%
Specificity %	81.6%	81.6%
False positive rate (%)	18.4%	18.4%
False negative rate (%)	22.9%	27.1%
*Low-risk P-II Strata*		
AUROC	0.77	0.73
True positive, *n*	16	17
False negative, *n*	39	38
False positive, *n*	10	14
True negative, *n*	135	131
Sensitivity %	29.1%	30.9%
Specificity %	93.1%	90.3%
False positive rate (%)	6.9%	9.7%
False negative rate (%)	70.9%	69.1%

Incorporated D1 SA-AKI, sTM, Angpt-2, Tie-2, and Angpt-2/Tie-2 ratio

### Classification and Regression Tree analyses yield an optimal model to predict D3 SA-AKI risk among subset of patients with high- or intermediate-PERSEVERE-II mortality risk strata

**Fig. 3:**
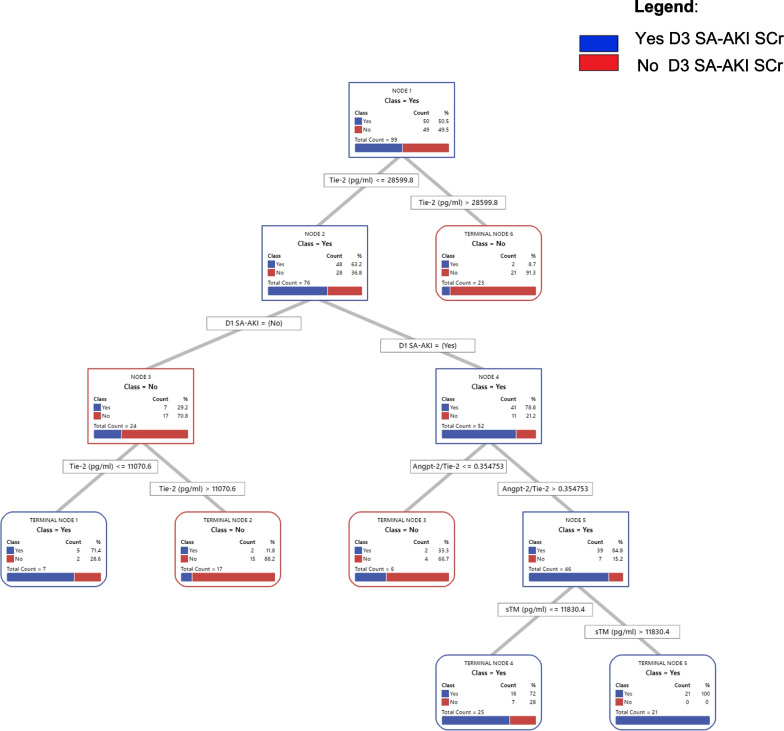
The PERSEVERENCE SA-AKI CART Model. Classification and regression analyses tree (CART) model to estimate risk of Day 3 sepsis-associated acute kidney injury based on serum creatinine criteria (D3 SA-AKI SCr) among patients with high- or intermediate-PERSEVERE-II mortality risk strata. The root node provides the total number of patients in the derivation cohort, and the number of those with and without D3 SA-AKI SCr, with the respective rates. Each daughter node provides the respective decision rule criterion and the number of those with and without D3 SA-AKI SCr, with the respective rates. The CART model had 6 terminal nodes (TN) which represent groups of patients who could not be separated further. Terminal nodes (TN) 1, 4, and 5 were deemed to have a high-risk of D3 SA-AKI SCr (≥ 71.4%); TN2, 3, and 6 were considered to have low-risk of D3 SA-AKI (< 11.8%), relative to rate of D3 SA-AKI Scr of 50.5% in the root note. Patients belonging to high or intermediate PERSEVERE-II mortality risk strata who had a Tie-2 concentration > 28,599 pg/mL (TN6) concentration had a low risk (8.7%) of D3 SA-AKI SCr. Among those with a Tie-2 concentrations ≤ 28,599 pg/mL, those without D1 SA-AKI could be further stratified once again based on Tie-2 concentrations; those with Tie-2 > 11,071 pg/mL had low-risk (11.8%) of D3 SA-AKI SCr (TN2) while those with Tie-2 ≤ 11,071 pg/mL had high-risk of D3 SA-AKI SCr (71.4%) (TN1). In contrast, those with Tie-2 ≤ 28,599 pg/mL and D1 SA-AKI, were further stratified based on Angpt-2/Tie-2 ratios. Patients with Angpt-2/Tie-2 < 0.35 had low-risk of D3 SA-AKI (33.3%, TN3). Those with high Angpt-2/Tie-2 ratios > 0.35 were further stratified based on sTM concentrations; those with sTM ≤ 11,830 pg/mL were high-risk of D3 SA-AKI SCr (72.0%, TN 4) while those with sTM > 11,820 pg/mL had a 100% risk of D3 SA-AKI SCr

Figure 3 shows the CART model to predict D3 SA-AKI SCr among patients with high- or intermediate-PERSEVERE-II mortality risk. Receiver operating characteristic curve and relative variable importance are shown in Additional file 4. Tie-2 concentration and Angpt-2/Tie-2 ratio were the most important predictor variables in this subset of patients. Terminal nodes (TN) 1, 4, and 5 were deemed to have a high-risk of D3 SA-AKI SCr (≥ 71.4%); TN2, 3, and 6 were considered to have low-risk of D3 SA-AKI SCr (< 11.8%). The AUROC for this newly derived “PERSEVERENCE SA-AKI” CART model was 0.90 and 0.77 upon tenfold cross-validation, with a sensitivity of 88% (95% CI 75–95%), specificity of 82% (95%CI 68–91%), positive predictive value of 83% (95% CI 70–91%), and negative predictive value of 87% (95% CI 73–95%) in the derivation cohort.

### Model performance in the validation cohort demonstrated reproducibility in identifying patients with high risk of D3 SA-AKI

Among the 224 patients in the validation cohort, 29 patients were categorized as high- and 55 patients were categorized as intermediate-PERSEVERE-II mortality risk. Classification of these patients (*n* = 84) in the validation cohort according to the PERSEVERENCE SA-AKI risk model is shown in Additional file 5. Among patients classified as low D3 SA-AKI risk, 29.0% (9/31) patients actually had D3 SA-AKI SCr. In contrast, among those categorized as high risk, 47.1% (25/53) had D3 SA-AKI. Finally, considering all 183 patients with high- or intermediate-PERSEVERE-II mortality risk in the derivation and validation cohorts, 64.7% (68/105) and 17.4% (15/78) patients categorized of high and low risk, according to the PERSEVERENCE SA-AKI model, actually had D3 SA-AKI SCr, as shown in Table in Additional file 5.

## Discussion

We present data on the association between markers of endothelial dysfunction and severe SA-AKI in a large cohort of critically ill children with septic shock. In our cohort, sTM, Angpt-2, and Tie-2 were independently associated with increased odds of D3 SA-AKI SCr. Further, endothelial dysfunction markers tested demonstrated variable response across PERSEVERE-II mortality risk strata, with Tie-2 concentrations and Angpt-2/Tie-2 ratios being influenced by the interaction between D3 SA-AKI SCr and PERSEVERE-II mortality risk strata. Subsequently, we derived and validated the performance of an endothelial biomarker-based model that predicted with high specificity the risk of D3 SA-AKI SCr among patients with a high- or intermediate-PERSEVERE-II mortality risk.

Several studies have reported on the association between endothelial dysfunction markers and risk of SA-AKI among adults. Single-center studies have identified the independent association of sTM, Angpt-2, and Angpt-1 with risk of AKI among critically ill adults, a vast majority of whom had sepsis as the inciting cause [12, 13]. Similar observations have been made among septic patients enrolled in the multi-center Finnish Acute Kidney Injury (FINNAKI) cohort, where both sTM and Angpt-2 were associated with an independent risk of 90-day mortality [14], and more recently among patients with severe sepsis and acute respiratory failure enrolled in the Validating Acute Lung Injury markers for Diagnosis (VALID) study where Angpt-2 outperformed other biomarkers in predicting risk of SA-AKI [15]. Importantly, the time point at which AKI was determined ranged from > 12 h up to 7 days from the time of ICU admission. Our data among critically ill children corroborate those among adults. The key distinction being our focus on D3 SA-AKI SCr, a clinically significant time point beyond which spontaneous recovery of kidney function is less likely and as such associated with poor outcomes [6, 11].

Recently, Bhatraju et al. [16] and Wiersma et al. [17] have identified subphenotypes of SA-AKI among critically ill adults with phenotypes AKI-SP2 and Subphenotype 2, respectively, demonstrating high levels of systemic inflammation and endothelial activation. In particular, AKI-SP2 was characterized by a high Angpt-2/Angpt-1 ratio—a key variable that helped distinguish subphenotypes [16]. Accordingly, the authors concluded that Angpt-2/Angpt-1 which serve to regulate microvascular barrier integrity may be linked to development of SA-AKI. Our data that low Tie-2 and high Angpt-2/Tie-2 ratios are highly predictive of D3 SA-AKI SCr among the most critically ill subset of pediatric septic shock, categorized as high- or intermediate-PERSEVERE-II mortality risk, are both novel and complementary to this literature among adults.

Our results may inform prognostic enrichment efforts to identify those at highest risk of SA-AKI among critically ill patients with septic shock [27]. Indeed, several of the endothelial biomarkers including sTM, Angpt-2, Tie-2, and Angpt-2/Tie-2 ratio were among the top predictor variables predictive of SA-AKI on day 7 in studies by our group that sought to develop models that have integrated PERSEVERE-endothelial biomarkers to develop a unified model to predict risk of multiple organ dysfunctions [20]. Future studies are required to determine whether such integrated models or alternatively sequential deployment of PERSEVERE-II followed by the endothelial-biomarker-based risk models in real time allow for identification of high-risk patients who may be amenable to targeted therapies.

The PERSEVERENCE SA-AKI CART prediction model detailed in this study has biologic plausibility. Tie-2 is an important molecule that plays a key role in stabilizing the endothelial barrier integrity and preventing capillary leak [28]. Angpt-2 antagonizes the effect of Tie-2 and serves to disrupt its function. Finally, thrombomodulin plays a vital role to inhibit coagulation pathway by serving as a co-factor in thrombin mediated activation of protein C. However, previous studies have demonstrated that Angpt-2 also binds and inhibits thrombomodulin function [29]. In concordance with these biological roles, patients with higher Tie-2 concentrations in our cohort had a low-risk of D3 SA-AKI SCr. Moreover, those with high Angpt-2/Tie-2 ratios and soluble thrombomodulin had a high-risk of D3 SA-AKI SCr. It is conceivable that among those patient with high Angpt-2/Tie-2 ratios had higher sTM concentrations in response to elevated Angpt-2. However, this is speculative given the observational nature of our study.

A randomized trial of recombinant human soluble thrombomodulin (rhTM) among 800 adult patients with sepsis-associated coagulopathy failed to demonstrate a 28-day all-cause mortality among critically ill patients [30]. More recently, in a retrospective single-center study in Japan among 97 adult patients with SA-AKI, rhTM administration was associated with lower 28-day mortality, improvement in renal function, and reduced use of renal replacement therapy at ICU discharge [31]. It remains plausible that the subset of patients identified by the PERSEVERENCE SA-AKI prediction model may have a biological predilection to respond to microvascular stabilizing therapies, including rhTM, that seek to restore the balance between Angpt-2/Tie-2 and improve endothelial barrier function. It is therefore conceivable that our model may be used to facilitate predictive enrichment in future clinical trials of such therapies among critically ill children with high risk of severe SA-AKI.

Our study has several limitations including (1) observational nature of study that precludes identification of causal mechanisms, (2) lack of baseline SCr and reliance on only SCr based definition of SA-AKI, (3) lack of urine output data, which may lead to underestimation of AKI, (4) relatively limited number of patients and consequently an inability to detect statistical differences in biomarkers between those with high- or intermediate-mortality risk, (5) consideration of a limited set of global rather than kidney specific markers of endothelial dysfunction that were selected based on current literature, (6) use of PERSEVERE-II based classification, which incorporates low platelets in the classification scheme and may therefore be correlated with endothelial dysfunction potentially biasing our results toward a positive association, and (7) the derivation cohort used to train the CART model represented those with a higher burden of organ dysfunctions, with consequent drop off in model performance when tested in the hold-out validation group who were less sick.

Despite these limitations, our data may inform future translational approaches to improving the care of critically ill children with SA-AKI. The strengths of our study include (1) a large cohort of pediatric septic shock patients, (2) unlike other black box machine learning algorithms, CART methodology provides clear biomarker thresholds based on which patients are divided into risk strata and can be used to test model performance in other populations, (3) fivefold cross-validation and internal validation of the model in a unique set of patients. Although the performance of the model was modest in validation group, our data represent real-world challenges to model performance when using biomarkers, including the potential for batch-to-batch variation in measurements.

## Conclusions

Biomarkers of endothelial dysfunction are independently associated with risk of severe sepsis-associated kidney injury and demonstrate variable responses across pediatric sepsis mortality (PERSEVERE-II) risk strata. Pending prospective internal and external validation, the risk prediction models developed herein may facilitate prognostic and predictive enrichment of critically ill children for selection in precision microvascular stabilizing therapies, which hold potential to meaningfully improve SA-AKI outcomes.

## Supplementary Information

**Additional file 1**. Clinical characteristics of patients according to PERSEVERE-II mortality class.

**Additional file 2**. Univariate associations between predictor variables and risk of D3 SA-AKI SCr.

**Additional file 3**: Box and whisker plots of concentrations of endothelial dysfunction markers among patients with and without Day 3 sepsis-associated acute kidney injury, across low-, intermediate-, and high PERSEVERE-II mortality risk strata. The asterisk indicates that the interaction between D3 SA-AKI and PERSEVERE-II mortality risk strata influenced concentrations of Tie-2 and Angpt-2/Tie-2 ratio.

**Additional file 4**:Top panel shows the receiver operating characteristic curve for the PERSEVERENCE SA-AKI CART model to estimate risk of Day 3 sepsis-associated acute kidney injury among patients with high- or intermediate-PERSEVERE-II mortality risk strata in training and test sets. It shows relative variable importance of predictor variables included in the model.

**Additional file 5**. Classification of patients with high- and intermediate- PERSEVERE-II mortality risk in the validation cohort (n=84) according to the PERSEVEREnce SA-AKI Risk model without any modifications.

**Additional file 6**. Online Supplement: R code for PERSEVERENCE CART Tree to predict D3 Sepsis Associated-Acute Kidney Injury among children with septic shock.

## Data Availability

All de-identified data including biomarker concentrations are available from the corresponding author upon reasonable request. The R code for the CART software is published in the Additional file 6.

## Abbreviations

IL-8: Interleukin 8
sTM: Soluble thrombomodulin
Angpt-1: Angiopoietin-1
Angpt-2: Angiopoietin-2
Tie-2: TEK tyrosine kinase
Angpt-2/Angpt-1: Angiopoietin-2/Angiopoietin-1 ratio
Angpt-2/Tie-2: Angiopoietin-2/Tie-2 ratio
ICAM-1: Intercellular adhesion molecule-1
VCAM-1: Vascular cell adhesion molecule-1
PECAM-1: Platelet endothelial cell adhesion molecule-1
