# Supplementary material for: Prognostic and predictive value of endothelial dysfunction biomarkers in sepsis-associated acute kidney injury: risk-stratified analysis from a prospective observational cohort of pediatric septic shock
Source: Crit Care. 2023 Jul 3;27:260. doi: 10.1186/s13054-023-04554-y (PMC10318688; doi:10.1186/s13054-023-04554-y)
Supplement: Supplementary file 1 — Additional file 1. Clinical characteristics of patients according to PERSEVERE-II mortality class. [file 13054_2023_4554_MOESM1_ESM.pdf]

Additional File 1.

Clinical characteristics of patients according to PERSEVERE-II mortality class

N=301	High risk (n=36)	Intermediate risk (n=63)	Low risk (n=202)	P value
Age (years)	2.4 (1.1, 5.2)	3.2 (0.7, 7.3)	3.3 (1.2, 6.8)	0.977
PRISM-III	22 (16, 28) *	14 (10, 18) *	10 (5, 14)	<0.001
P-II Mort. Prob	0.398 (0.391, 0.407) **	0.187 (0.181, 0.193) *	0.009 (0.005, 0.012)	<0.001
28-day mortality	11 (30.6 %) **	9 (14.3 %) *	3 (1.5 %)	<0.001
Complicated course	23 (63.9 %) **	30 (47.6 %) *	60 (29.7 %)	<0.001
D1 SA-AKI	25 (69.4 %) **	37 (58.7 %) *	64 (31.8 %)	<0.001
D3 SA-AKI	21 (58.3 %) **	29 (46.1 %) *	56 (27.7 %)	<0.001
D7 SA-AKI	19 (52.8 %) **	22 (34.9 %) *	45 (22.3 %)	<0.001
Day 1 RRT	9 (25.0 %) **	6 (9.5 %) *	4 (2.0 %)	<0.001
Day 3 RRT	13 (36.1 %) **	9 (14.3 %) *	7 (3.5 %)	<0.001
Day 7 RRT	17 (47.2 %) **	11 (17.5 %) *	9 (4.5%)	<0.001

**Indicates significant difference relative to intermediate- and low-risk strata after adjusting for multiple comparisons.

*Indicates significant difference relative to low-risk strata alone after adjusting for multiple comparisons.
