# Supplementary material for: Prognostic and predictive value of endothelial dysfunction biomarkers in sepsis-associated acute kidney injury: risk-stratified analysis from a prospective observational cohort of pediatric septic shock
Source: Crit Care. 2023 Jul 3;27:260. doi: 10.1186/s13054-023-04554-y (PMC10318688; doi:10.1186/s13054-023-04554-y)
Supplement: Supplementary file 2 — Additional file 2. Univariate associations between predictor variables and risk of D3 SA-AKI SCr. [file 13054_2023_4554_MOESM2_ESM.pdf]

Additional File 2.

Univariate associations between predictor variables and risk of D3 SA-AKI SCr

Variable	OR	P value
Age	0.94 (0.90, 0.99)	0.019
Sex (Female)	0.8 (0.5, 1.2)	0.274
PRISM-III	1.04 (1.01, 1.06)	0.001
P-II Mortality Probability X10	1.4 (1.12, 1.7)	<0.001
sTM (Log10)	56.4 (18.4, 173.2)	<0.001
Angpt-1 (Log10)	0.4 (0.2, 0.6)	<0.001
Angpt-2 (Log10)	7.4 (3.7, 14.3)	<0.001
Tie-2 (Log10)	0.2 (0.1, 0.6)	0.003
Angpt-2/Angpt-1	1.07 (1.01, 1.14)	0.015
Angpt-2/Tie-2	2.6 (1.8, 3.7)	<0.001
VCAM-1 (Log10)	3.9 (2.0, 7.9)	<0.001
ICAM-1 (Log10)	14.7 (4.8, 43.7)	<0.001
PECAM-1 (Log10)	1.6 (0.6, 4.1)	0.343
