# Supplementary material for: Prognostic and predictive value of endothelial dysfunction biomarkers in sepsis-associated acute kidney injury: risk-stratified analysis from a prospective observational cohort of pediatric septic shock
Source: Crit Care. 2023 Jul 3;27:260. doi: 10.1186/s13054-023-04554-y (PMC10318688; doi:10.1186/s13054-023-04554-y)
Supplement: Supplementary file 3 — Additional file 3: Box and whisker plots of concentrations of endothelial dysfunction markers among patients with and without Day 3 sepsis-associated acute kidney injury, across low-, intermediate-, and high PERSEVERE-II mortality risk strata. The asterisk indicates that the interaction between D3 SA-AKI and PERSEVERE-II mortality risk strata influenced concentrations of Tie-2 and Angpt-2/Tie-2 ratio. [file 13054_2023_4554_MOESM3_ESM.pdf]

Additional File 3.

General linear models for association between markers of endothelial dysfunction, D3 SA-AKI SCr and PERSEVERE-II strata.

Biomarker	Term	Coeff. (SE)	P value
sTM (log10)	D3 SA-AKI	0.129 (0.03)	<0.001
	P-II Strata		
	High	0.169 (0.06)	0.003
	Intermediate	0.116 (0.04)	0.003
	D3 SA-AKI X P-II Strata		
	D3 SA-AKI and High	0.064 (0.07)	0.402
	D3 SA-AKI and Intermediate	-0.023 (0.06)	0.698
Angpt-1 (log10)	D3 SA-AKI	-0.118 (0.06)	0.049
	P-II Strata		
	High	-0.468 (0.11)	<0.001
	Intermediate	-0.003 (0.07)	0.966
	D3 SA-AKI X P-II Strata		
	D3 SA-AKI and High	0.066 (0.14)	0.646
	D3 SA-AKI and Intermediate	-0.185 (0.11)	0.106
Angpt-2 (log10)	D3 SA-AKI	0.204 (0.05)	<0.001
	P-II Strata		
	High	0.375 (0.09)	<0.001
	Intermediate	0.285 (0.06)	<0.001
	D3 SA-AKI X P-II Strata		
	D3 SA-AKI and High	-0.098 (0.13)	0.441
	D3 SA-AKI and Intermediate	-0.036 (0.10)	0.721
Tie-2 (log10)	D3 SA-AKI	0.054 (0.03)	0.089
	P-II Strata		
	High	0.086 (0.05)	0.117
	Intermediate	0.013 (0.04)	0.730
	D3 SA-AKI X P-II Strata		
	D3 SA-AKI and High	-0.311 (0.07)	<0.001
	D3 SA-AKI and Intermediate	-0.215 (0.06)	<0.001
Angpt-2/Angpt-1	D3 SA-AKI	1.037 (0.52)	0.047
	P-II Strata		
	High	3.499 (0.92)	<0.001
	Intermediate	0.521 (0.63)	0.413
	D3 SA-AKI X P-II Strata		
	D3 SA-AKI and High	1.090 (1.25)	0.383
	D3 SA-AKI and Intermediate	0.389 (0.99)	0.694
Angpt-2/Tie-2	D3 SA-AKI	0.147 (0.12)	0.238
	P-II Strata		

	High	0.322 (0.22)	0.132
	Intermediate	0.463 (0.15)	0.002
	D3 SA-AKI X P-II Strata		
	D3 SA-AKI and High	0.657 (0.30)	0.027
	D3 SA-AKI and Intermediate	0.664 (0.23)	0.005
VCAM-1	D3 SA-AKI	0.08 (0.05)	0.070
	P-II Strata		
	High	0.257 (0.08)	0.002
	Intermediate	0.093 (0.05)	0.102
	D3 SA-AKI X P-II Strata		
	D3 SA-AKI and High	-0.072 (0.12)	0.523
	D3 SA-AKI and Intermediate	0.008 (0.08)	0.929
ICAM-1	D3 SA-AKI	0.116 (0.03)	0.001
	P-II Strata		
	High	0.202 (0.05)	0.001
	Intermediate	0.144 (0.04)	0.001
	D3 SA-AKI X P-II Strata		
	D3 SA-AKI and High	-0.04 (0.08)	0.622
	D3 SA-AKI and Intermediate	-0.09 (0.06)	0.129
PECAM-1	D3 SA-AKI	0.013 (0.06)	0.701
	P-II Strata		
	High	0.009 (0.06)	0.874
	Intermediate	0.086 (0.04)	0.043
	D3 SA-AKI X P-II Strata		
	D3 SA-AKI and High	0.017 (0.08)	0.838
	D3 SA-AKI and Intermediate	-0.06 (0.06)	0.292

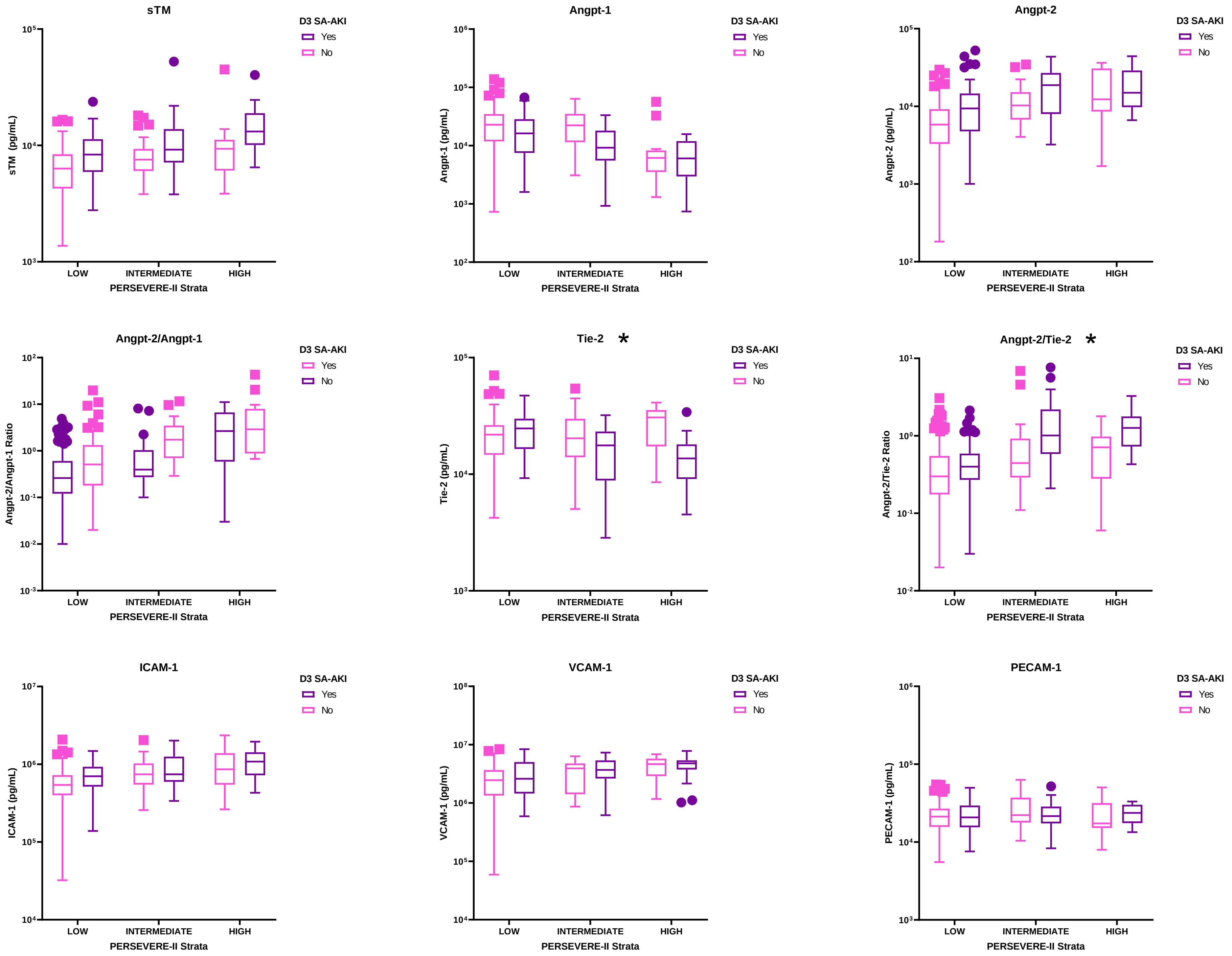
sTM
sTM (pg/mL)
D3 SA-AKI
Yes
No
Angpt-1
Angpt-1 (pg/mL)
D3 SA-AKI
Yes
No
Angpt-2
Angpt-2 (pg/mL)
D3 SA-AKI
Yes
No
Angpt-2/Angpt-1
Angpt-2/Angpt-1 Ratio
D3 SA-AKI
Yes
No
Tie-2 *
Tie-2 (pg/mL)
D3 SA-AKI
Yes
No
Angpt-2/Tie-2 *
Angpt-2/Tie-2 Ratio
D3 SA-AKI
Yes
No
ICAM-1
ICAM-1 (pg/mL)
D3 SA-AKI
Yes
No
VCAM-1
VCAM-1 (pg/mL)
D3 SA-AKI
Yes
No
PECAM-1
PECAM-1 (pg/mL)
D3 SA-AKI
Yes
No
LOW
INTERMEDIATE
HIGH
PERSEVERE-II Strata
