# Supplementary material for: Prognostic and predictive value of endothelial dysfunction biomarkers in sepsis-associated acute kidney injury: risk-stratified analysis from a prospective observational cohort of pediatric septic shock
Source: Crit Care. 2023 Jul 3;27:260. doi: 10.1186/s13054-023-04554-y (PMC10318688; doi:10.1186/s13054-023-04554-y)
Supplement: Supplementary file 4 — Additional file 4:Top panel shows the receiver operating characteristic curve for the PERSEVERENCE SA-AKI CART model to estimate risk of Day 3 sepsis-associated acute kidney injury among patients with high- or intermediate-PERSEVERE-II mortality risk strata in training and test sets. It shows relative variable importance of predictor variables included in the model. [file 13054_2023_4554_MOESM4_ESM.pdf]

Additional File 4.

a.

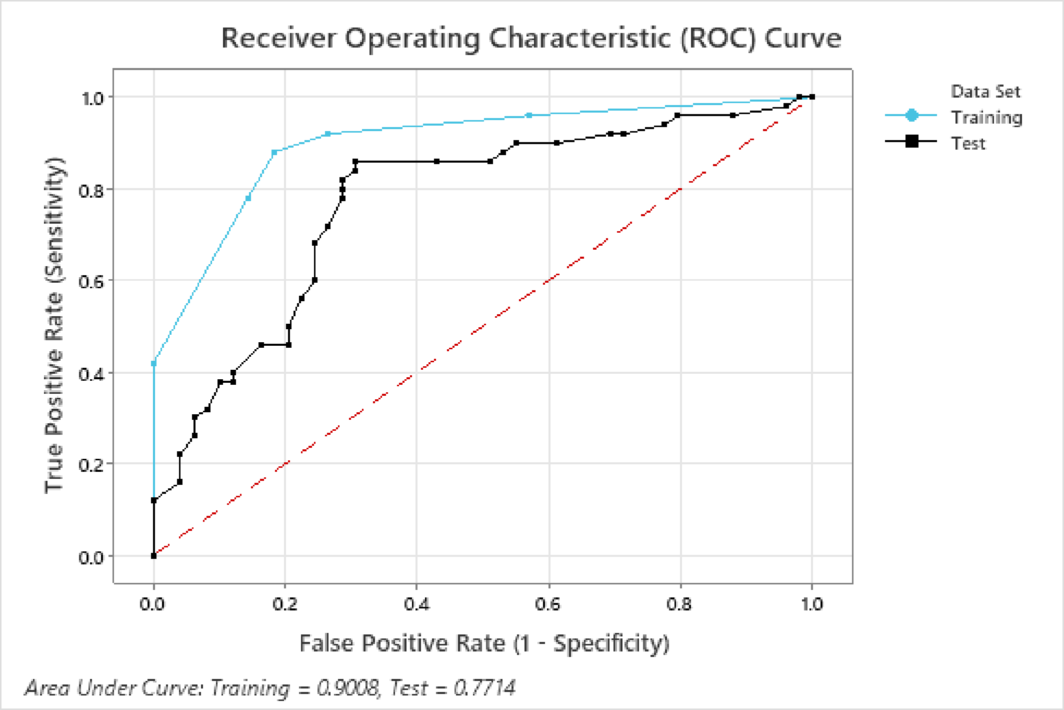

b.

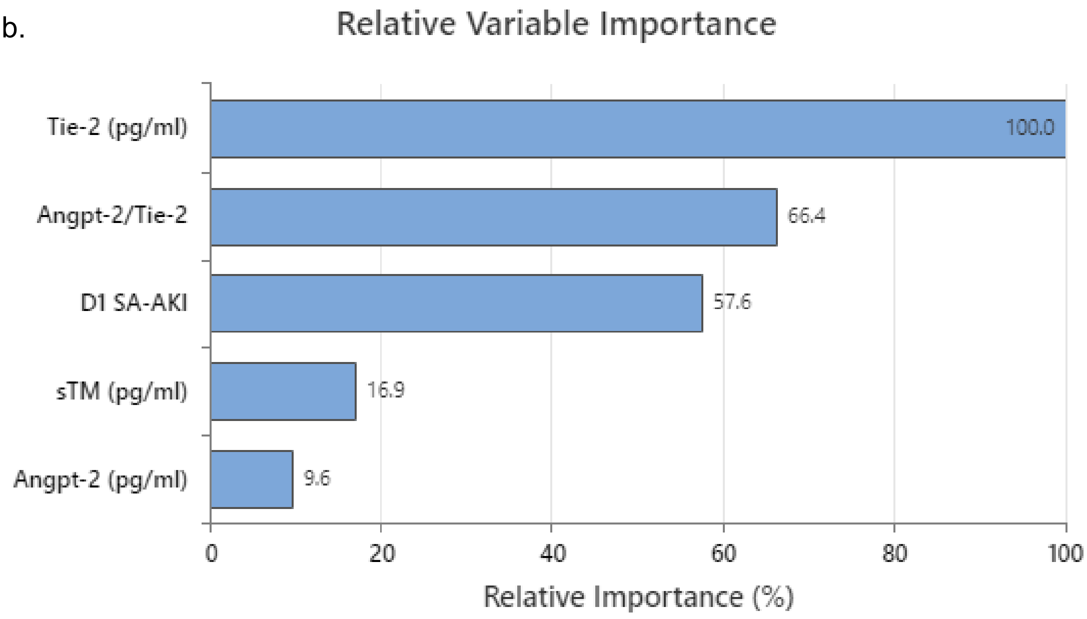

Variable importance measures model improvement when splits are made on a predictor. Relative importance is defined as % improvement with respect to the top predictor.
