# Supplementary material for: Prognostic and predictive value of endothelial dysfunction biomarkers in sepsis-associated acute kidney injury: risk-stratified analysis from a prospective observational cohort of pediatric septic shock
Source: Crit Care. 2023 Jul 3;27:260. doi: 10.1186/s13054-023-04554-y (PMC10318688; doi:10.1186/s13054-023-04554-y)
Supplement: Supplementary file 5 — Additional file 5. Classification of patients with high- and intermediate- PERSEVERE-II mortality risk in the validation cohort (n=84) according to the PERSEVEREnce SA-AKI Risk model without any modifications. [file 13054_2023_4554_MOESM5_ESM.pdf]

Additional File 5.

Figure 1.

Classification of patients with high- and intermediate- PERSEVERE-II mortality risk in the validation cohort (n=84) according to the PERSEVEREnce SA-AKI Risk model without any modifications.

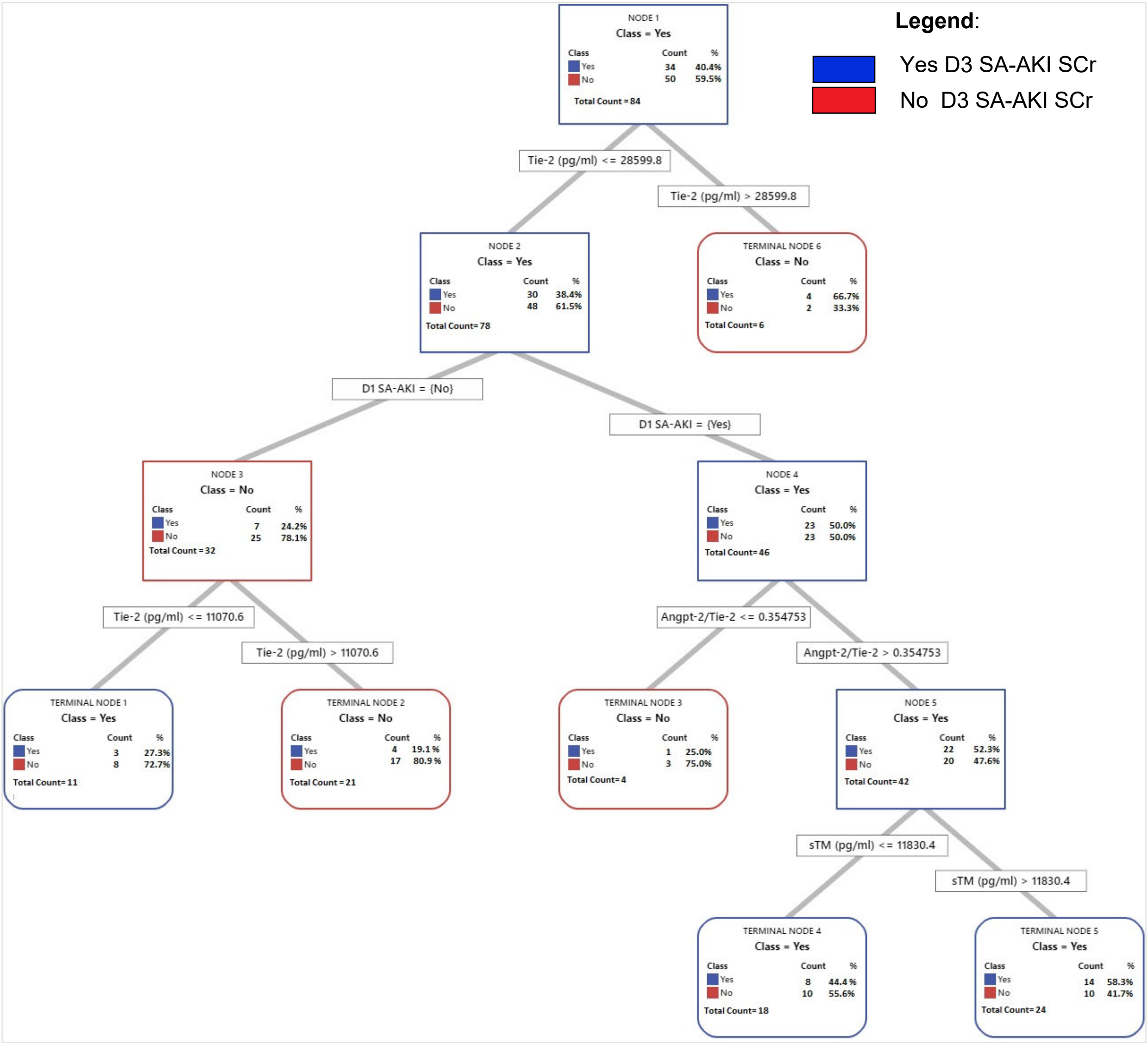

Table. 1

Demographic characteristics and clinical outcomes among patients categorized as high- vs. low D3 SA-AKI risk in the combined derivation and validation cohorts among those categorized as having high- or intermediate- PERSEVERE-II mortality risk.

Variable	High D3 SA-AKI risk (n=105)	Low D3 SA-AKI risk (n=78)	p value
Age (Years)	4 (1, 9)	4 (1, 9)	0.724
Sex, Female (%)	51 (48.5%)	29 (37.2%)	0.193
Race (Self-identified)			0.640
White/Caucasian	77 (73%)	57 (73%)	
Black/African American	13 (13%)	6 (8%)	
Other	14 (13%)	11 (14%)	
Ethnicity			0.114
Hispanic or Latino	11 (11%)	14 (11%)	
PRISM-III	16 (10, 24)	12 (8, 19)	0.030
28-day Mortality	28 (26.7%)	11 (14.1%)	0.055
Complicated course	66 (62.8%)	26 (33.3%)	<0.001
PICU LOS	8 (4, 15)	7 (3, 15)	0.085
PICU Free Days	20 (13, 24)	21 (13, 25)	0.086
D3 SA-AKI	68 (64.7%)	15 (19.2%)	<0.001
Day 7 CRRT*	25 (33.3%)	2 (4.6%)	<0.001

*Adjusted for study mortality before day 7 and those patients transferred out of ICU without persistent SA-AKI.
