# Supplementary material for: Prognostic and predictive value of endothelial dysfunction biomarkers in sepsis-associated acute kidney injury: risk-stratified analysis from a prospective observational cohort of pediatric septic shock
Source: Crit Care. 2023 Jul 3;27:260. doi: 10.1186/s13054-023-04554-y (PMC10318688; doi:10.1186/s13054-023-04554-y)
Supplement: Supplementary file 6 — Additional file 6. Online Supplement: R code for PERSEVERENCE CART Tree to predict D3 Sepsis Associated-Acute Kidney Injury among children with septic shock. [file 13054_2023_4554_MOESM6_ESM.pdf]

Online Supplement:

Title: Prognostic and Predictive Value of Endothelial Dysfunction Biomarkers in Sepsis-Associated Acute Kidney Injury: Risk Stratified Analysis from a Prospective Observational Cohort of Pediatric Septic Shock.

Authors: Mihir R. Atreya[1, 2]; Natalie Z. Cvijanovich[3]; Julie C. Fitzgerald[4]; Scott L. Weiss[4]; Michael T. Bigham[5]; Parag N. Jain[6]; Adam J. Schwarz[7]; Riad Lutfi[8]; Jeffrey Nowak[9]; Geoffrey L. Allen[10]; Neal J. Thomas[11]; Jocelyn R. Grunwell[12]; Torrey Baines[13]; Michael Quasney[14]; Bereketeab Haileselassie[15]; Matthew N. Alder[1, 2]; Stuart L. Goldstein[2,16], and Natalja L. Stanski[1, 2].

Author Affiliations:

1. Division of Critical Care Medicine, Cincinnati Children's Hospital Medical Center and Cincinnati Children's Research Foundation, Cincinnati, 45229, OH, USA.
2. Department of Pediatrics, University of Cincinnati College of Medicine, Cincinnati, OH 45267, USA.
3. UCSF Benioff Children’s Hospital Oakland, Oakland, CA 94609, USA.
4. Children’s Hospital of Philadelphia, Philadelphia, PA 19104, USA.
5. Akron Children’s Hospital, Akron, OH 44308, USA.
6. Texas Children’s Hospital and Baylor College of Medicine, Houston, TX 77030, USA.
7. Children’s Hospital of Orange County, Orange, CA 92868, USA.
8. Riley Hospital for Children, Indianapolis, IN 46202, USA.
9. Children’s Hospital and Clinics of Minnesota, Minneapolis, MN 55404, USA.
10. Children’s Mercy Hospital, Kansas City, MO 64108, USA.
11. Penn State Hershey Children’s Hospital, Hershey, PA 17033, USA.
12. Children’s Healthcare of Atlanta at Egleston, Atlanta, GA 30322, USA.
13. University of Florida Health Shands Children’s Hospital, Gainesville, FL 32610, USA.
14. CS Mott Children’s Hospital at the University of Michigan, Ann Arbor, MI 48109, USA.
15. Lucile Packard Children’s Hospital Stanford, Palo Alto, CA 94304, USA.
16. Division of Nephrology, Cincinnati Children's Hospital Medical Center and Cincinnati Children's Research Foundation, Cincinnati, 45229, OH, USA.

Corresponding author:
Mihir R. Atreya, MD, MPH
Cincinnati Children’s Hospital Medical Center
Division of Critical Care Medicine, MLC2005
3333 Burnet Avenue
Cincinnati, OH, 45229, USA
Email: Mihir.Atreya@cchmc.org

R code for PERSEVERENCE CART Tree to predict D3 Sepsis Associated-Acute Kidney Injury among children with septic shock.

```
Install.package(dplyr)
library(dplyr)

#load in you CSV file
data <- ***

#add the terminal node designations

data <- data %>% mutate(Terminal_Node =
 case_when(
data$`Tie-2` <= 28599 & data$`D1 SAAKI`<= 0 & data$`Tie-2` <= 11070 ~ "TN1",
data$`Tie-2` <= 28599 & data$`D1 SAAKI`<= 0 & data$`Tie-2` > 11070 ~ "TN2",
data$`Tie-2` <= 28599 & data$`D1 SAAKI`<= 1 & data$`Ang-2/Tie-2` <= 0.35~"TN3",
data$`Tie-2` <= 28599 & data$`D1 SAAKI`>= 1 & data$`Ang-2/Tie-2` > 0.35 & data$`sTM` <=
11830 ~ "TN4",
data$`Tie-2` <= 28599 & data$`D1 SAAKI`>= 1 & data$`Ang-2/Tie-2` > 0.35 & data$`sTM` >
11830 ~ "TN5",
data$`Tie-2` > 28599 ~ "TN6"))

#now add the D3 SA-AKI risk

data <- data %>% mutate(D3_SAAKI_Prob=
                case_when(data$Terminal_Node == "TN1" ~ "0.714",
                    data$Terminal_Node == "TN2" ~ "0.118",
                    data$Terminal_Node == "TN3" ~ "0.333",
                    data$Terminal_Node == "TN4" ~ "0.720",
                    data$Terminal_Node == "TN5" ~ "1.000",
                    data$Terminal_Node == "TN6" ~ "0.087",))


#now you can add the mortality risk class.
data <- data %>% mutate(D3_SAAKI_PREDICTED_Class =
                case_when(data$D3_SAAKI_Prob >= 0.714 ~ "HIGH",
                TRUE ~ "LOW"))
```
